# Successful Use of Hyperbaric Oxygen as Adjunctive Therapy for a Nonhealing Venous Ulcer in a Patient with Systemic Sclerosis and Pulmonary Arterial Hypertension: A Case Report and Review of the Literature

**DOI:** 10.1155/2020/4750375

**Published:** 2020-02-24

**Authors:** Isaac Biney, Tina Dudney, Mitchell Goldman, Lee Carder, Elise Schriver

**Affiliations:** ^1^Department of Medicine, Division of Pulmonary Disease and Critical Care Medicine, University of Tennessee Graduate School of Medicine, Knoxville, TN, USA; ^2^Department of Surgery, University of Tennessee Graduate School of Medicine, Knoxville, TN, USA

## Abstract

Skin ulcers are a common complication of systemic sclerosis (SSc) that can significantly impact the quality of life. There have been recent reports on the use of hyperbaric oxygen therapy (HBO_2_T) in the management of nonhealing systemic sclerosis skin ulcers. The effect of HBO_2_T on pulmonary arterial hypertension (PAH), another common and potentially life-threatening complication of SSc, is unclear with literature on the subject lacking. We present the case of a 65-year-old female with limited SSc complicated by severe PAH and a nonhealing left lower extremity venous ulcer. HBO_2_T was successfully used as an adjunct in her management resulting in complete resolution of the venous ulcer and improved quality of life without any adverse effects on her pulmonary arterial hypertension.

## 1. Introduction

Systemic sclerosis (SSc) is a chronic connective tissue disease with multiorgan involvement characterized by diffuse microangiopathy and the deposition of collagen into the skin and internal organs leading to progressive fibrosis. It is a subtype of a heterogeneous group of autoimmune diseases referred to as scleroderma and can be subcategorized by the degree of skin and internal organ fibrosis into limited SSc and diffuse SSc [1]. Skin ulcers are recorded in up to 50% of patients with SSc, and pathogenic mechanisms include microvascular damage and vasospasm [2]. Vascular impairment may also cause nonhealing of ulcers arising due to other mechanisms. These ulcers can be very painful and can cause a significant decline in a patient's quality of life. Management of these ulcers can be very challenging, and therapeutic guidelines remain controversial [2].

Hyperbaric oxygen therapy (HBO_2_T) involves the administration of 100% oxygen in a closed chamber pressurized to greater than 1 atmosphere absolute (ATA). Its use in ischemic and nonhealing wounds has been documented since the mid-20^th^ century, and there are well-established indications [3]. Its benefits for these indications have been attributed to antihypoxic, antimicrobial, antiedema, anti-inflammatory, and angiogenetic effects [4]. Recently, there have been several case reports of successful use of HBO_2_T in nonhealing ulcers, involving both the digits and extremities, in patients with SSc [4–6].

One relatively common and harmful complication of SSc is pulmonary arterial hypertension (PAH). Prevalence rates up to 19% have been reported in the literature and it is stated as the leading cause of death in this population [7–12]. There have been concerns raised by experts about the safety of HBO_2_T in patients with significant PAH. In a recent study looking at HBO_2_T-treated ulcers in SSc subjects, patients with moderate to severe pulmonary hypertension were excluded [13]. There is currently no literature evaluating effects of HBO_2_T in patients with PAH.

We report a case of successful use of HBO_2_T as adjunctive therapy for the treatment of a nonhealing venous ulcer in a patient with limited SSc complicated by PAH.

## 2. Case Presentation

A 65-year-old female was diagnosed with limited systemic sclerosis 11 years previously after presenting with Raynaud's phenomenon complicated by digital necrosis resulting in amputation of the tip of her left third finger and interstitial lung disease. Her SSc had been managed with various disease-modifying agents, but each had to be discontinued due to intolerance. She was maintained on chronic low-dose prednisone. Her first encounter with our facility was when she presented to vascular surgery with chronic venous insufficiency and a nonhealing venous ulcer on the medial aspect of her left lower leg which had developed in October 2016. At the time, her functional status had declined from being fully functional to being confined to a wheelchair or a walker due to the discomfort from her leg ulcer and exertional dyspnea. She had debridement of the wound and radiofrequency ablation of her left saphenous vein and developed hypotension following the procedure requiring ICU admission. During workup for the hypotension, she had an echocardiogram which showed a left ventricular ejection fraction (LVEF) of 30-35%, a severely dilated right ventricle (RV) with moderately reduced function as measured by a tricuspid annular plane systolic excursion (TAPSE) of 1.1 cm and an estimated right ventricular systolic pressure of 66 mmHg. A noncontrast CT scan of her chest showed mild interstitial lung disease. She subsequently had a right heart catheterization which showed a pulmonary artery pressure of 91/37 mmHg with a mean of 55, a transpulmonary gradient of 47 mmHg, a pulmonary capillary wedge pressure of 8 mmHg, and a pulmonary vascular resistance of 18.1 Woods units. Her cardiac output was 2.6 L/min and cardiac index was 1.8 L/min/m^2^. She had a normal ventilation perfusion scan. She was diagnosed with PAH with a World Health Organization (WHO) functional class of IV. A repeat echocardiogram prior to the initiation PAH therapy showed a LVEF of 55-60%. The patient refused parenteral prostanoids. She was treated with sildenafil and macitentan and inhaled treprostinil with improvement of her WHO functional class to class III.

**Figure 1:**
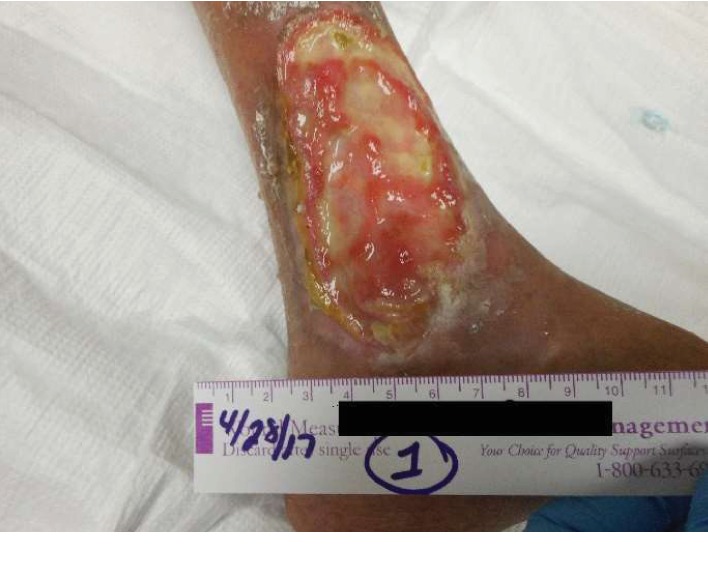
Left lower extremity venous ulcer at the time of initial presentation to the wound care center.

**Figure 2:**
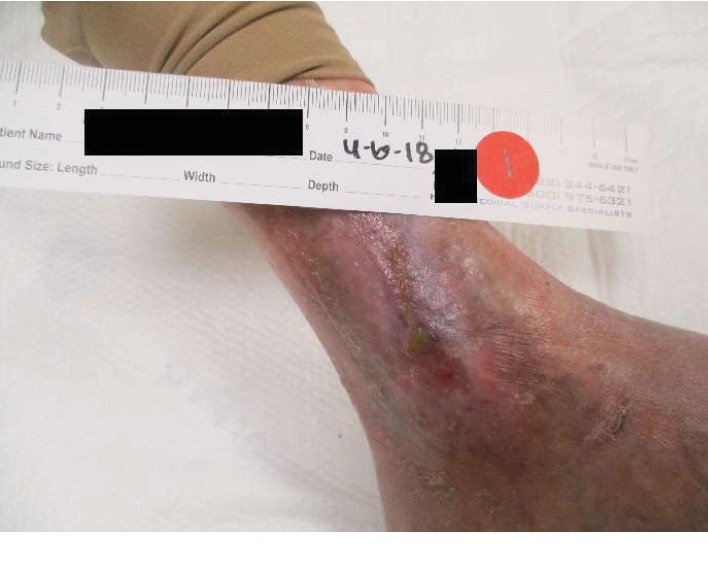
Left lower extremity demonstrating complete resolution of venous ulcer after a year of treatment.

She began treatment of her left lower extremity wound at our wound care center in April 2017. At her initial evaluation, her wound measured 10 cm long, 5.3 cm wide, and 0.3 cm deep with an area of 41.626 cm^2^ and a volume of 12.488 cm^3^. It was described as a full-thickness wound without exposed structures, with well-defined borders, a large amount of serosanguineous exudate and with medium amount of granulation tissue (Figure 1). Initial treatment was with nonadherent Silvercel as the primary dressing and Kerlix with ABD pad applied as the secondary dressing. After 2 months, the primary dressing was changed to the bioengineered skin substitute Apligraf and Mepitel One. Three months later, it was switched to Adaptic. The maximum reduction in wound area achieved with these modalities was 78.1% reduction after 7 months of treatment. The patient was thought to have exhausted her options at this point. After a thorough search of the literature, consulting with the patient's pulmonologist and cardiologist, and detailed discussion with the patient outlining the potential risks involved and possible benefits, a joint decision was made to attempt HBO_2_T which started in November 2017. Her most recent echocardiogram had been obtained 4 months prior, and it showed an LVEF of 61%, grade 1 diastolic dysfunction, a moderately dilated RV with mildly reduced function, TAPSE 1.6 cm, and an estimated RVSP of 72 mmHg. The patient received 30 treatments between 11/13/2017 and 12/19/2017 in a monoplace chamber at 2 ATA with 90 minutes of oxygen with no air breaks. Duration of each treatment ranged from 106 to 110 minutes. No immediate adverse events were noted during any of the treatment sessions, and she did not experience any worsening of her exercise tolerance. After completion of HBO_2_T, wound care continued with Apligraf, Adaptic, and Xeroform. Complete wound closure was achieved by April 2018 (Figure 2). She had 3 wound debridements during her treatment at the wound care center, 2 while receiving HBO_2_T. At follow-up, her functional status has improved to WHO class II, echocardiogram showed a LVEF of 65-70%, mild left atrial enlargement, grade II diastolic dysfunction, mild right atrial enlargement, and a mildly to moderately dilated right ventricle with normal function measured by a TAPSE of 1.9 cm. She had started exercising on a stationary bicycle and worked 5-7 hours per day. Prior to HBO_2_T, significant nocturnal wound pain interfered with her sleep. After wound closure, her pain resolved and quality of life vastly improved. The patient continues to do well. At her most recent follow-up in October 2019, her functional status remains at WHO class II, serum brain natriuretic peptide level was 45 pg/mL, and her echocardiogram showed a normal-sized RV with normal function and an RVSP of 33.9 mmHg.

## 3. Discussion

Ulcers related to SSc (SSc-SU) have been known to be a challenging manifestation of the disease, often difficult to heal and recurrent. Their management has improved over the last decade with a multidisciplinary approach involving the combined use of systemic and advanced local treatments but has still been described as suboptimal [2]. Though our patient had a venous ulcer, similar challenges can be encountered in a patient with SSc as the disease has been associated with poor wound healing on account of the vasculopathy.

There has recently been developing interest in the use of HBO_2_T in patients with SSc with intractable ulcers with several published case reports demonstrating its success. In our patient, HBO_2_T was successfully used as an adjunct for treatment of her nonhealing ulcer [4–6]. Some experts have expressed concerns about the use of HBO_2_T in patients with PAH. Whether this is a generalized opinion or not, there have been no human studies evaluating this or reports of adverse events of HBO_2_T when used in patients with PAH. A recent study found that patients with SSc complicated by skin ulcers were more likely to have increased pulmonary arterial pressures [2]. Most studies looking at ulcers in systemic sclerosis have focused on digital ulcers with very little attention to nonhealing lower extremity ulcers [14]. Development of PAH and right ventricular dysfunction are likely to add to the challenge of managing such ulcers. In a study evaluating lower extremity ulcers in patients with SSc, 10 out of 249 patients had active nondigital leg ulcers. Of these, one patient had PAH and venous insufficiency and was among the 3 patients who had a nonhealing ulcer [14]. It is reasonable to deduce that patients with PAH will form a significant proportion of patients with SSc-SU who get to the point of being considered for HBO_2_T if indeed HBO_2_T becomes a standard adjunctive therapy in intractable SSc-SU.

There is scant literature evaluating the effect of HBO_2_T on the pulmonary circulation in humans. Pulmonary oxygen toxicity manifesting as an acute exudative phase and subacute proliferative phase occurs with increasing fraction of inspired oxygen and partial pressure of oxygen. However, current applications of HBO_2_T have not been shown to cause pulmonary symptoms or clinically significant pulmonary function defects [15]. Weaver et al. reported pulmonary artery catheter data in 10 normal subjects exposed to 40 minutes of hyperbaric air followed by 40 minutes of HBO_2_T. Pulmonary vascular resistance (PVR) decreased by up to 48% during HBO_2_T with reduction in pulmonary artery pressure (PAP) by up to 19%. Measurements returned to baseline after removal of hyperbaric conditions [16]. Long-term effects are unknown. In a study evaluating the effect of hyperoxia on the pulmonary circulation in rats, pulmonary vascular remodeling with altered hemodynamics including an increase in PVR and PAP was observed after continuous exposure to hyperoxia for 7 days at normobaric pressure [17]. Jacobson et al. demonstrated the development of pulmonary hypertension in rabbits after hyperbaric exposure to 100% oxygen for 1 hour with evidence to suggest that this was due to increased synthesis of thromboxane [18]. Armstrong et al. evaluated the safety of HBO_2_T in SSc patients. Two patients were said to have not received HBO_2_T on account of moderate to severe pulmonary hypertension, the underlying reason for this was not discussed. There have been case reports associating HBO_2_T with pulmonary edema in patients with left-sided heart failure [19, 20]. Possible mechanisms attributed to this finding include increased peripheral vasoconstriction leading to increased cardiac afterload, increased oxidative myocardial stress, decreased left ventricular (LV) compliance by oxygen radical-mediated reduction in nitric oxide right and left ventricular imbalance, and increased pulmonary capillary permeability. A study of the effect of HBO_2_T on cardiac performance in anesthetized dogs showed increased systemic vascular resistance but no change in PVR, and the data suggested that HBO_2_T may act by a differential effect on the autonomic innervation of the right and left ventricles [21].

Our successful use of HBO_2_T as adjunctive therapy in our patient suggests that it may be safe to use in patients with PAH, but further research is needed to fully evaluate this as the risk benefit is unknown. With increasing interest in HBO_2_T use in SSc-SU and with the high prevalence of PAH in this population, it will be important to not automatically exclude patients who could benefit from a potentially life-changing therapy.
